# Editorial: Birth Advantages and Relative Age Effects

**DOI:** 10.3389/fspor.2021.721704

**Published:** 2021-07-23

**Authors:** Adam L. Kelly, Jean Côté, David J. Hancock, Jennifer Turnnidge

**Affiliations:** ^1^Birmingham City University, Birmingham, United Kingdom; ^2^Queen's University, Kingston, ON, Canada; ^3^Memorial University of Newfoundland, St. John's, NL, Canada

**Keywords:** RAEs, youth sport, athlete development, talent identification, talent development

We are delighted to have this Research Topic on birth advantages and relative age effects come to fruition. In 2019, the editorial team had several informal conversations about birth advantages and relative age effects in sport. A recurring theme in those discussions was that much of the existing research in the field used archival methods to collect data, and few researchers integrated theory into their studies. As a result, the editorial team met in the autumn of 2019 and formalized our Research Topic for *Frontiers*. Subsequently, we solicited submissions that: (1) focused on birth advantages of any kind, (2) went beyond mining birthdates from registration databases, and (3) were theoretically-driven. Examining the articles included in this Research Topic, we believe these objectives were met, and are incredibly grateful to all of the authors for their strong contributions. The editorial team also made a concerted effort to recruit diverse authors who could support this Research Topic. In doing so, we aimed to highlight varied research approaches, ensuring a unique contribution to the field of birth advantages in sport. The 18 articles that appear in this Research Topic were penned by 65 authors, many of whom are internationally-recognized scholars in this field. These authors represent 35 universities or sport institutions (e.g., professional sport teams or national governing bodies) spanning 13 countries. Owing to our approach, it is not surprising that 16 sports are studied within this Research Topic, with 11 studies including female athletes as participants. Ultimately, we believe that the diverse authorship and samples have resulted in a unique Research Topic, which has significantly advanced the field through its various approaches that should facilitate thoughtful discussion about birth advantages.

In 1983, Paula and Rodger Barnsley first discovered relative age effects in sport as they read a program at an elite amateur ice hockey game that listed all the participating players' birthdates (Barnsley et al., 1985). Understandably then, the first explorations of relative age effects in sport were not a result of applying existing theory to a new setting. For decades, however, birth advantage and relative age effect studies tended to outline these phenomena in various contexts, with little attention paid to understanding and predicting the effects through theory. It was not until 2013 when a theoretical model was proposed to understand relative age effects. The first model was offered by Hancock et al. (2013), which focused on the role social agents (i.e., parents, coaches, and athletes) played in creating and perpetuating relative age effects. Shortly thereafter, Wattie et al. (2015) suggested a constraints-based model (i.e., individual, environment, and task constraints) to explain relative age effects. We believe that grounding research in theory is imperative to advancing this field, which is why we explicitly stated in our call for proposals that the research be theory-driven. Throughout the submission and review process, we encouraged authors to strengthen their theoretical connections. Ultimately, this was successful as the authors of the manuscripts in this Research Topic included theories to support their research. We hope that as this field grows, researchers continue to use existing or new theories to shape their research.

As noted above, birth advantage and relative age effect studies have relied on archival data to show associations between some relatively unchangeable variables (e.g., birthdate, birthplace, handedness, gender, or maturity status) and some outcome measures of achievement (e.g., member of an elite team in sport or performance indicators). In the last few years, more diverse research approaches and methodologies have been used to document the “why” and “how” of the relationship between birth characteristics and athlete development. For example, social relationships have been proposed as a primary mechanism that adjusts the strength of the relationships between birth characteristics and outcomes. Moving forward, it appears that more studies driven by strong theoretical frameworks are needed to provide a better understanding of the mediating mechanisms that affect birth advantages in sport. Additionally, research on birth advantages would benefit from studies based on exploratory research questions such as “When do birth advantages disappear?” and “For whom does age and anthropometric banding work?” The various articles in this Research Topic provide examples of innovative qualitative (e.g., content analysis, composite narratives, and questionnaires) and quantitative approaches (e.g., Bayesian analysis, correlation and regression analysis, and multivariate analysis of covariance) that are starting to be used in birth advantages research. It is our hope that this Research Topic will inspire researchers to move beyond descriptive studies of relative age effects and continue to design new studies with innovative methodologies that will shed light on the “why,” “how,” “when,” and “for whom” of birth advantages in sport.

Our purpose for this Research Topic was to explore the organizational structures in youth sport that contribute to birth advantages and relative age effects. The contributing authors embraced this opportunity, and through their inclusion of a variety of research topics, have advanced our knowledge of various organizational and social structures that influence birth advantages and relative age effects. As an example, although maturity status inevitably interacts with relative age, Doncaster et al. used FC Barcelona as a case study to encourage researchers and stakeholders (i.e., coaches, practitioners, and policy makers) to appreciate factors beyond the physical (e.g., technical, psychological, and perceptual-cognitive attributes) when examining relative age effects. Moreover, Kelly et al. revealed that those born toward the end of the cut-off date were almost four times more likely to achieve senior professional and international levels in English rugby union compared to those born at the beginning of the cut-off date. They suggest that relatively younger players might have a greater likelihood of achieving expertise following entry into a talent pathway due to benefitting from more competitive play against relatively older peers during their development (e.g., the underdog hypothesis; Gibbs et al., 2012). In addition, Smith et al. showed how “confusion reigned” following the change of cut-off dates in 2015 by the US Soccer Federation, demonstrating a possible disconnect between research and youth sport policy with regards to possible solutions for relative age effects, which can lead to unintended consequences for key stakeholders in sport. Overall, it is essential that researchers and stakeholders use this information to develop an awareness of the integrated organizational and social environments that shape relative age and birth advantage effects in sport.

To gain a deeper understanding of birth advantages and relative age effects in sport, it is important to recognize that they often represent a by-product of sport organizations' policies regarding grouping athletes by chronological age (Turnnidge and Kelly, 2021). Although such policies are often intended to promote developmentally appropriate levels of challenge and create more equal competition, it is evident that these policies can have unintended consequences. Indeed, despite their widespread prevalence, there appears to be a paucity of empirical research and practical application of strategies specifically designed to moderate birth advantages and relative age effects [see Webdale et al. (2020) for a review]. Thus, an additional aim of our Research Topic was to encourage submissions that examined contemporary strategies that address birth advantages and relative age effects. As an individual sport example, Kelly et al. offered a new approach named “birthday-banding,” whereby athletes move up to their next birthdate group on their birthday, with the aim of removing particular selection time-points and fixed chronological age groups. From a team sport perspective, Helsen et al. proposed “estimated development age” as a new method for grouping young athletes using the midway point of their chronological and developmental birthdates, with the aim of decreasing relative age *and* maturity-related biases. As we navigate our way toward a new era of birth advantage and relative age literature, we encourage researchers and practitioners to work collaboratively to design, implement, and evaluate a range of fresh, theoretically-driven solutions. By doing so, we can help moderate birth advantages and relative age effects while continuing to promote positive participation, performance, and personal development outcomes in youth sport across the immediate, short-, and long-term timescales (Côté et al., 2021).

It was a pleasure to assemble this Research Topic. We hope the reader finds the articles included to be informative, innovative, and inspiring. Thank you to all the contributing authors and reviewers, without whom, this Research Topic would not be possible.

## Author Contributions

All authors listed have made a substantial, direct and intellectual contribution to the work, and approved it for publication.

## Conflict of Interest

The authors declare that the research was conducted in the absence of any commercial or financial relationships that could be construed as a potential conflict of interest.

## Publisher's Note

All claims expressed in this article are solely those of the authors and do not necessarily represent those of their affiliated organizations, or those of the publisher, the editors and the reviewers. Any product that may be evaluated in this article, or claim that may be made by its manufacturer, is not guaranteed or endorsed by the publisher.
